# Association Patterns of Ontological Features Signify Electronic Health Records in Liver Cancer

**DOI:** 10.1155/2017/6493016

**Published:** 2017-08-06

**Authors:** Lawrence W. C. Chan, S. C. Cesar Wong, Choo Chiap Chiau, Tak-Ming Chan, Liang Tao, Jinghan Feng, Keith W. H. Chiu

**Affiliations:** ^1^Department of Health Technology and Informatics, Hong Kong Polytechnic University, Hung Hom, Hong Kong; ^2^Philips Research China, Shanghai, China; ^3^Department of Diagnostic Radiology, University of Hong Kong, Pok Fu Lam, Hong Kong

## Abstract

Electronic Health Record (EHR) system enables clinical decision support. In this study, a set of 112 abdominal computed tomography imaging examination reports, consisting of 59 cases of hepatocellular carcinoma (HCC) or liver metastases (so-called HCC group for simplicity) and 53 cases with no abnormality detected (NAD group), were collected from four hospitals in Hong Kong. We extracted terms related to liver cancer from the reports and mapped them to ontological features using Systematized Nomenclature of Medicine (SNOMED) Clinical Terms (CT). The primary predictor panel was formed by these ontological features. Association levels between every two features in the HCC and NAD groups were quantified using Pearson's correlation coefficient. The HCC group reveals a distinct association pattern that signifies liver cancer and provides clinical decision support for suspected cases, motivating the inclusion of new features to form the augmented predictor panel. Logistic regression analysis with stepwise forward procedure was applied to the primary and augmented predictor sets, respectively. The obtained model with the new features attained 84.7% sensitivity and 88.4% overall accuracy in distinguishing HCC from NAD cases, which were significantly improved when compared with that without the new features.

## 1. Introduction

Sheer amount of clinical data hosted by the electronic health record (EHR) system facilitates the exploration of disease signatures and potentiates the relevant clinical decision support functions [1, 2].

As a real-time, digital patient-centered record, EHR contains a large amount of patient information and laboratory and test results. It provides opportunities to enhance patient care, to embed performance measures in clinical practice, and to make information available instantly and securely to the authorized users [3]. These voluminous complex data contain abundant input for precision medicine and big data analytics, which can extract huge knowledge to improve the quality of healthcare [4]. Integrated exploitation of multiple heterogeneous sources also serves for multidisciplinary renovation like biomedical engineering. In this article, extrapolating EHRs' human lexical judgments from computational models of semantics is one of the approaches that can minimize human intervention and save human efforts significantly.

The rapid development of EHR provides good opportunity to utilize the data for risk modeling and clinical decisions. Besides the well-structured demographics and laboratory information, clinical reports in EHR provide great potential for machine learning and data mining to exploit the detailed clinical information to improve risk modeling and prediction. For example, machine learning approaches could be developed based on admission notes and progress notes to improve prediction of major adverse cardiac events (MACE) of acute coronary syndrome (ACS) [5, 6]. Extraction of key information from reports is a foundation step to enable these data mining applications.

As a simplifying representation in natural language processing and information retrieval, the bag-of-words model has long been applied in the text clustering tasks, in which documents are represented by independently treated single terms [7]. Without a reference terminology, a bag of words can be extracted from a document to form an array of unique features whose weights are determined by the term frequencies and form the feature vector. However, the length of feature vector increases monotonically with the number of documents in the dataset of interest, jeopardizing the practicality of the bag-of-words model.

Recently, some researchers focused on the application of ontology for extracting the conceptual features from documents. Based on reference ontology, the feature vectors consist of common fixed elements, which have already been defined before the feature extraction. Such ontological feature vector model could improve the performance of text retrieval and classification [8, 9]. In some studies, feature vector model has been developed for converting the clinical texts and image patterns of an EHR into an array of numerical values [10–13].

The support of a medical ontology is required to map textual information, such as image findings in a diagnostic report, to a feature vector [12, 13]. Systematized Nomenclature of Medicine (SNOMED) Clinical Terms (CT) is an ontological standard of clinical terms, which are organized as concepts and linked with “is-a” or inverse “is-a” relationships [14–17]. In such hierarchical structure, concepts at a particular level could be chosen as the feature concepts.

Some studies have compared SNOMED-CT with other standards, such as International Classification of Diseases (ICD) and MEDCIN [18, 19]. As a trigger to order laboratory tests, clinical conditions were extracted from laboratory guidelines and mapped to ICD10 and SNOMED-CT. It was found that ICD10 could cover 43.1% of clinical conditions only, whereas 80.1% of these conditions were mapped by SNOMED-CT. For representing traumatic brain injury (TBI) concepts, SNOMED-CT yielded a sensitivity of 90%, outperforming MEDCIN whose sensitivity was 49%. Thus, SNOMED-CT was selected as the reference ontology in this study.

**Figure 1:**
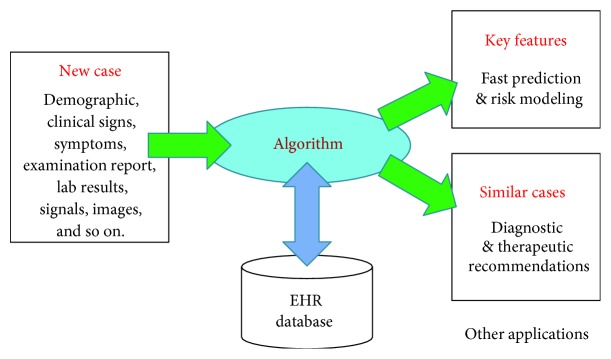
Clinical decision support application of EHR similarity algorithm.

The semantic distance between a clinical term in EHR and a feature concept can be quantified by counting the edges along the path connecting them in the “is-a” hierarchy [10–12, 20, 21]. Aggregating all the semantic distances to the feature concepts generates an ontological feature vector that characterizes an EHR with its disease context. A study has performed the evaluation and comparison between information content and edge counting approaches proposed by various published works against benchmarks [11]. It was found that features built with edge-counting outperformed most of the information content approaches. Therefore, the edge counting is necessary for weighting the features. We hypothesize that the feature association patterns derived from the EHRs can uniquely distinguish a disease group from the nondisease group. If such distinguishable association patterns exist, new features could be derived from the patterns and incorporated into the existing ontological feature vector to strengthen the ontological characterization of EHRs and thus the classification performance using similarity algorithm, as illustrated in Figure 1.

The identified ontological patterns can be used to develop a clinical decision support functions. For the new cases, similar cases retrieved from EHR database using the patterns provide clinicians with evidence of the feasible diagnostic and therapeutic options. The similarity search algorithm based on the ontological vector model has been successfully applied to similar radiological image report retrieval and similar radiotherapy treatment plan retrieval [22–24].

In addition to the clinical evidence, the association between concepts in the patterns can be used to remind a clinician of checking the inclusion of a concept when its associated concept has already been mentioned in an EHR.

## 2. Methods

### 2.1. Data Collection

We collected retrospectively 112 image reports of abdominal computed tomography examinations from the radiology departments of four local hospitals in Hong Kong. HCC or liver metastases were found in 59 cases (called HCC group for simplicity) and the other 53 cases had no abnormality detected (NAD group). These 112 cases were randomly selected from the pool of image reports where HCC or liver metastases were reported in the diagnoses of HCC cases and not reported in the diagnoses of NAD cases. Before the data collection, third party clinical personnel have removed the patient name, identity card number, telephone number, and address from the reports and assigned a randomly generated unique ID to each case. We have obtained Human Subject Ethics Approval from the Hong Kong Polytechnic University (HSEARS20140710002).

### 2.2. Ontological Feature Extraction

The HCC-related clinical terms were extracted manually from the image reports according to SNOMED-CT curated in the Unified Medical Language System (UMLS; license code: NLM-0315126310). During the extraction process, the whole image reports were read and interpreted. The negation and uncertainty of a disease, disorder, or image finding was regarded as “not detected” and the corresponding term was not considered in the ontological feature mapping. Modifiers for clinical terms were not found in the image reports. To facilitate the future studies on a bigger dataset, the extraction can be automatic if the terms in image reports have been already tagged by SNOMED-CT or extracted automatically by text-mining methods. UMLS organizes clinical terms in concepts, and SNOMED-CT defines the relationship between concepts using the “is-a” hierarchical tree. The extracted terms were projected to the feature concepts at a particular level to ensure consistent comparison between reports.

In our previous study, a set of EHRs were collected from 47 subjects of type II diabetic patients in Hong Kong [21]. Levels 1–4 of the SNOMED-CT hierarchy were considered as the individual candidate sets of the feature concepts. For each level, ontological feature vectors were generated using the alignment with SNOMED-CT hierarchy and the similarity score between every possible pair of EHRs was calculated. Using SNOMOD-CT level 4, the accuracy was highest for ranking the agreement of carotid plaque identification in EHR pairs. It is important to note that level 4 has already had 6964 feature terms, providing sufficient granularity for characterizing EHRs. The use of level 5 is indeed infeasible due to the tremendously large number of features. Due to the optimal classification granularity, level 4 concepts were considered as feature concepts in this work.

**Figure 2:**
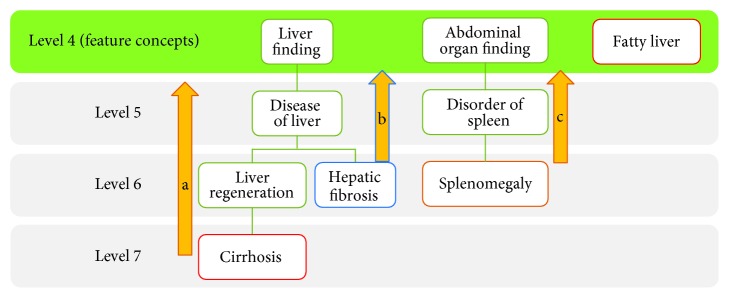
Edge counting based on level 4 concepts: “liver finding,” “abdominal organ finding,” and “fatty liver.” (a) “Cirrhosis” at level 7 is the descendant of “liver finding,” edge count is 3. (b) Edge count between “hepatic fibrosis” and “liver finding” is 2. (c) “Splenomegaly” is the descendant of “abdominal organ finding” but not “liver finding.” Thus, edge count of “splenomegaly” with “abdominal organ finding” is 2 and that with “liver finding” is infinity. “Fatty liver” is a feature concept, and thus, the edge count with itself is 0. Diagram was extracted from [22].

Edge-counting approach is illustrated in Figure 2. For each report, the ontological features, [*a*_1_, *a*_2_,…, *a*_*m*_], were generated using edge-counting approach based on the following formula:

(1) $$\begin{array}{c} a_{i}=\frac{\sqrt{p_{i}}}{1+\min_{j=1,\ldots ,n}s_{ij}}, \end{array}$$

where *p*_*i*_ is the conditional probability of the *ith* feature concept given the occurrence of liver cancer and *s*_*ij*_ represents the edge count between the *i*th feature concept and the *j*th clinical term extracted from a report. A smaller edge count means that the feature concept is conceptually closer to the clinical term. Therefore, the minimum of the edge counts should be taken to determine the degree of activation of a feature concept. PubMed document clustering has been successfully demonstrated using the edge-counting method [25].

With the value between 0 and 1, *a*_*i*_ indicates the relevance between the *i*th feature concept and a clinical term in a report. Such relevance can be modulated by the conditional probability, *p*_*i*_, which is estimated by the specific term-weighting approach [22]. Indeed, a similarity measure derived from direction cosine represents the sum of the product of ontological features. Each product of corresponding features eliminates the square root, and the value *p*_*i*_ becomes the weight associated with the product of the degree of feature concept activation between two EHRs.

It is obvious that the values of *a*_*i*_ follow a nonnormal distribution in the HCC and NAD populations, which violates the assumption of statistical analysis using Pearson correlation coefficient. Rank-based inverse normalization is a popular approach that converts the feature values to those normally distributed across individuals [26]. Those features with zero values do not cause any effect on the characterization of image reports and the association patterns between features. Thus, those zero-valued features were excluded in inverse normalization process and remain unchanged. For each feature concept, the nonzero values of a_*i*_ were ranked by *R*_*i*_ ∈ [1, *N* − *N*_0_] among reports of the group where *N* and *N*_0_ are, respectively, the total number of reports and the number of zero-valued features in the group. The activation value of the *i*th feature concept is given by

(2) $$\begin{array}{c} z_{i}=\Phi^{-1}\left(\frac{R_{i}-\xi }{N-N_{0}-2\xi +1}\right), \end{array}$$

where *Φ*^−1^ represents the standard normal quantile function and *ξ* denotes a constant, whose value is given by zero as suggested by van der Waerden [27]. The activation values of a feature concept across a group form the following vector:

(3) $$\begin{array}{c} u_{i}={\left[z_{i}\left(1\right),z_{i}\left(2\right),\ldots ,z_{i}\left(N\right)\right]}^{T}. \end{array}$$

Note that nonzero *z*_i_(*k*) follows normal distribution, *N*(0, 1), after inverse normalization.

### 2.3. Ontological Association Patterns

The association level between two feature concepts was denoted by *C*_*d*_(*i*, *j*) for the HCC group and *C*_*n*_(*i*, *j*) for the NAD group, as given by the following formulas:

(4) $$\begin{array}{c} C_{d}\left(i,j\right)=\;|\;r\left(u_{di},u_{dj}\right)\;|\;=\left|\frac{1}{N_{d}}\overset{N_{d}}{\underset{k=1}{\sum }}z_{di}\left(k\right)z_{dj}\left(k\right)\right|\\ C_{n}\left(i,j\right)=\;|\;r\left(u_{ni},u_{nj}\right)\;|\;=\left|\frac{1}{N_{n}}\overset{N_{n}}{\underset{k=1}{\sum }}z_{ni}\left(k\right)z_{nj}\left(k\right)\right|, \end{array}$$

where *u*_*di*_ and *u*_*dj*_ represent the vectors weighting the *ith* and *jth* feature concepts across the HCC group; *u*_*ni*_ and *u*_*nj*_ represent the vector weighting the *ith* and *jth* feature concepts across the NAD group; and *r*(*u*_*i*_, *u*_*j*_) is Pearson correlation coefficient between two arrays. Two sets of correlation coefficients, *C*_*d*_ and *C*_*n*_, in the HCC and NAD groups formed two cumulative distributions, *F*_*d*_ and *F*_*n*_, which were compared using two-sample Kolmogorov-Smirnov (KS). To test the significant difference, the maximum deviation between two cumulative distributions, *D* value, was compared with its critical value, *D*_*α*_, which is derived based on our developed method [28] and given by following equations. A correlation threshold, at which *F*_*d*_ and *F*_*n*_ were extremely deviated, can be identified and used to characterize the perturbed ontological association pattern.

(5) $$\begin{array}{c} D=\underset{C}{\max}\;|\;F_{d}\;\left(C\right)-F_{n}\;\left(C\right)\;|\;\\ F_{d}\;\left(C\right)=prob\;\left(C_{d}\;\leq C\right)\\ F_{n}\;\left(C\right)=prob\;\left(C_{n}\;\leq C\right)\\ D_{\alpha }=\gamma \left(\alpha \right)\sqrt{\frac{4}{m\left(m-1\right)}}, \end{array}$$

where *α* is the significance level, that is, 0.05, *γ*(0.05) = 3.1, and *k* = 30 in this study. The critical value of *D* is 0.2102, which has been proved by exhaustive computer simulations [28].

### 2.4. New Features Derived from Association Patterns

It is interesting to explore some new features, which signify the image reports of HCC cases, based on the above-mentioned ontological association patterns. The first new feature, *z*_1_′(*k*), is the square of the sum of activation values characterizing the image report of the *k*th case in a group.

(6) $$\begin{array}{c} z_{1}^{\prime }\left(k\right)={\left(\overset{m}{\underset{i=1}{\sum }}z_{i}\left(k\right)\right)}^{2}. \end{array}$$

The expected value of this new feature can be estimated by its average over the group.

(7) $$\begin{array}{c} \frac{1}{N}\overset{N}{\underset{k=1}{\sum }}z_{1}^{\prime }\left(k\right)=\frac{1}{N}\overset{N}{\underset{k=1}{\sum }}{\left(\overset{m}{\underset{i=1}{\sum }}z_{i}\left(k\right)\right)}^{2}=\frac{1}{N}\overset{N}{\underset{k=1}{\sum }}\left(\overset{m}{\underset{i=1}{\sum }}{\left(z_{i}\left(k\right)\right)}^{2}+2\overset{i=m,j=m}{\underset{i\neq j,i=1,j=1}{\sum }}z_{i}\left(k\right)z_{j}\left(k\right)\right)=m+2\overset{i=m,j=m}{\underset{i\neq j,i=1,j=1}{\sum }}\frac{1}{N}\overset{N}{\underset{k=1}{\sum }}z_{i}\left(k\right)z_{j}\left(k\right)\leq m+2\overset{i=m,j=m}{\underset{i\neq j,i=1,j=1}{\sum }}C\left(i,j\right). \end{array}$$

It is clearly shown that the expected value of this new feature forms the lower bound of the sum of association levels over all possible pairs of features in the group. The second new feature, *z*_2_′(*k*), is the square of the sum of the absolute values of activation values characterizing the image report of the *k*th case in a group.

(8) $$\begin{array}{c} z_{2}^{\prime }\left(k\right)={\left(\overset{m}{\underset{i=1}{\sum }}\left|z_{i}\left(k\right)\right|\right)}^{2}. \end{array}$$

The expected value of the second new feature, again, can be estimated by its average over the group.

(9) $$\begin{array}{c} \frac{1}{N}\overset{N}{\underset{k=1}{\sum }}z_{2}^{\prime }\left(k\right)=\frac{1}{N}\overset{N}{\underset{k=1}{\sum }}{\left(\overset{m}{\underset{i=1}{\sum }}\left|z_{i}\left(k\right)\right|\right)}^{2}=\frac{1}{N}\overset{N}{\underset{k=1}{\sum }}\left(\overset{m}{\underset{i=1}{\sum }}{\left(z_{i}\left(k\right)\right)}^{2}+2\overset{i=m,j=m}{\underset{i\neq j,i=1,j=1}{\sum }}\left|z_{i}\left(k\right)z_{j}\left(k\right)\right|\right)=m+2\overset{i=m,j=m}{\underset{i\neq j,i=1,j=1}{\sum }}\frac{1}{N}\overset{N}{\underset{k=1}{\sum }}\left|z_{i}\left(k\right)z_{j}\left(k\right)\right|\geq m+2\overset{i=m,j=m}{\underset{i\neq j,i=1,j=1}{\sum }}C\left(i,j\right). \end{array}$$

The above formula clearly shows that the expected value of the second new feature defines the upper bound of the sum of association levels over all possible pairs of features in the group. When the KS test indicates that the ontological association patterns of two groups are significantly different, we expect that the sum of association levels of a group is distinguishable from that of the other group. Therefore, the new features could signify the difference between two groups.

### 2.5. Logistic Regression

The statistical analysis was performed by SPSS (IBM SPSS Statistics 22; Armonk, NY). Binary logistic regression selects and estimates the optimal subset of independent variables for predicting categorical outcome *Y* coded by 1 or 0, which represents HCC and NAD in this work. Stepwise forward procedure was used to obtain the logistic regression model where the potential predictors were prioritized and entered into the model one by one until the predictive power was optimized. The procedure results in the following model with *M* predictors,

(10) $$\begin{array}{c} logit=\ln\left(\frac{P}{1-P}\right)=\beta_{0}+\beta_{1}X_{1}+\beta_{2}X_{2}+\cdots +\beta_{M}X_{M}, \end{array}$$

where logit is the estimated log odds of *Y* = 1, *P* is the estimated probability of *Y* = 1, *X*_*i*_ is the *ith* predictor entered into the models, and *i* is the coefficient associated with the *ith* predictor for *i* = 1,…, *k*. The statistical significance of the association between the outcome and each predictor is indicated by *p* < 0.05. For a well-balanced sample, we assume 50% of the cases will be classified as *Y* = 1 and the cut-off of logit is set at 0. Sample is imbalanced when the number of cases with an outcome category is about 2–5 times that with the other category. For an imbalanced sample, the constant 0 is corrected by deducting the log odds of *Y* = 1 observed in the sample. Omnibus test of model coefficients indicate the overall performance of an identified model.

Two sets of candidate predictors, primary set and augmented set, are considered for identifying the logistic regression models. The primary set consists of the activation values of feature concepts: {*z*_1_(*k*), *z*_2_(*k*),…, *z*_*m*_(*k*)}. The augmented set is composed of the activation values of feature concepts and three new features derived from the association patterns: {*z*_1_(*k*), *z*_2_(*k*),…, *z*_*m*_(*k*), *z*_1_′(*k*), *z*_2_′(*k*)}.

### 2.6. Experimental Settings

**Figure 3:**
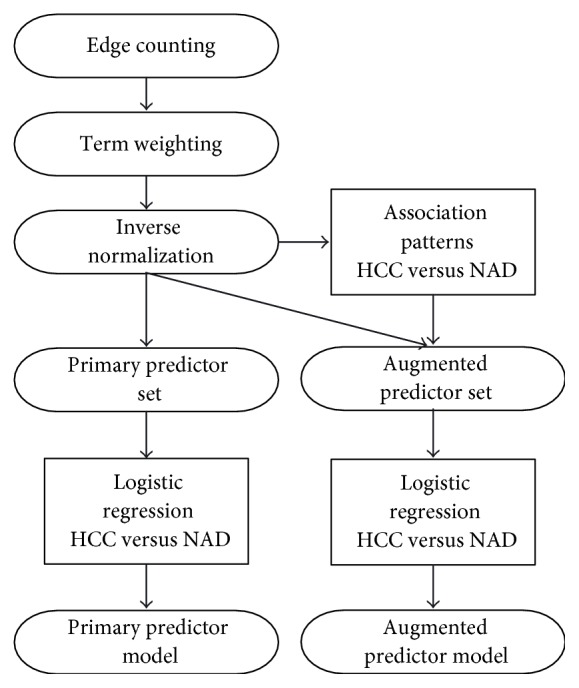
Flow chart of the performed experimental steps.

Figure 3 illustrates the flow chart of the experimental steps performed in the study.

### 2.7. Performance Evaluation

Sensitivity, specificity, and overall accuracy were used to evaluate the performance of two logistic regression models based on the primary and augmented predictor sets. To examine the agreement between primary predictor model (PPM) and augmented predictor model (APM), 2 × 2 contingency tables for HCC, NAD, and all cases are constructed. The McNemar test is used to compare sensitivities, specificities, and overall accuracy of two models. The difference in performance is considered significant if the *P* value is less than 0.05.

## 3. Results

### 3.1. Extracted Features

From 59 and 53 image reports of respective HCC and NAD groups, 38 clinical terms were extracted and mapped to 38 unique concepts in UMLS. Based on the approach illustrated in Figure 2, these terms were then projected to 30 feature concepts at level 4 of SNOMED-CT “is-a” hierarchy (Table 1). After counting the edges and estimating the conditional probabilities of these concepts, their weightings were calculated and formed 30 × 59 and 30 × 53 matrices for HCC and NAD groups.

### 3.2. Ontological Association Patterns

**Figure 4:**
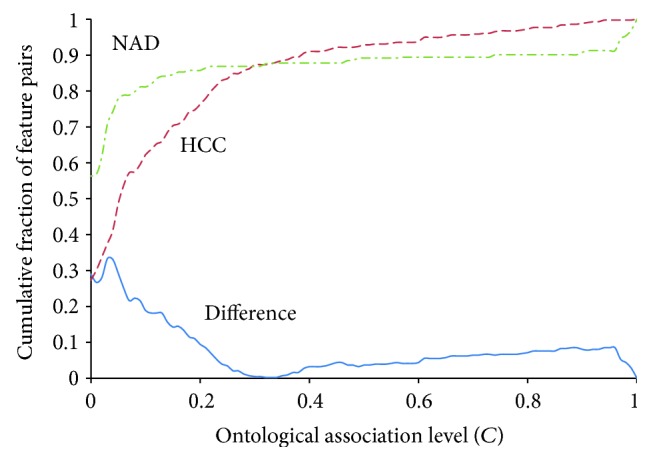
Two distinct ontological association patterns. Cumulative distributions of ontological association levels across NAD and HCC groups are indicated by dash-dotted and dash lines, respectively. Solid line represents the difference between these two cumulative distributions.

The association level between every two feature concepts was calculated. We generated 435 association levels for each of HCC and NAD groups.  Figure 4 shows the cumulative distributions of association levels for the two groups and their difference. The maximum deviation, *D* = 0.333, was found at *C* = 0.03 and greater than its critical value. Therefore, the two ontological association patterns are significantly different.

### 3.3. Primary Predictor Model

**Figure 5:**
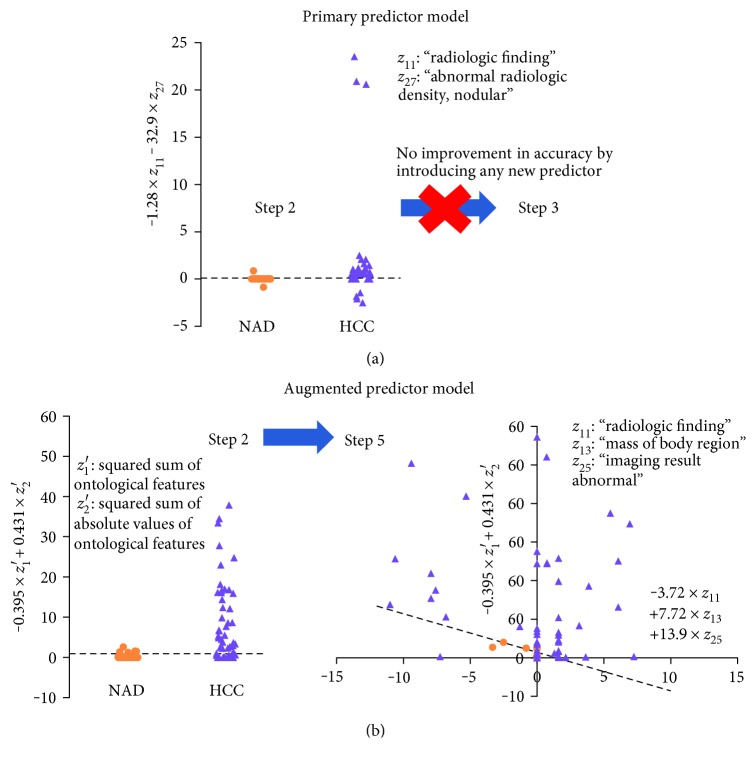
Logistic regression classification results using primary and augmented predictor models. (a) Primary predictor model (PPM): the identification procedure incorporated *z*_11_, “radiologic finding,” *z*_27_, and “abnormal radiologic density, nodular” in the first two steps. The process stopped at step 2 as new predictor cannot make any improvement in classification. (b) Augmented predictor model (APM): two new features, *z*_1_′ and *z*_2_′, were included in the model in the first two steps of the procedure. The predictors, *z*_1_′ and *z*_2_′, represent the squared sums of ontological features and their absolute values, respectively. The identification proceeds to step 5 that extends the feature space into three predictor dimensions, *z*_11_, *z*_13_, and *z*_25_, representing “radiologic finding,” “mass of body region,” and “imaging result abnormal.”

The stepwise forward procedure stops at step 2 where the prediction accuracy is optimal, yielding the following regression:

(11) $$\begin{array}{c} logit=-1.28{\;z}_{11}-32.9{\;z}_{27}-0.114, \end{array}$$

where *z*_11_ represents the activation value of “radiologic finding” and *z*_27_, “abnormal radiologic density, nodular.” The predictor *z*_11_, “radiologic finding,” is significantly associated with the log-odds of HCC (*p* = 0.016). The constant has been adjusted to compensate the imbalanced NAD and HCC cases. Omnibus test shows that the variance of log-odds explained by the model is significantly greater than the unexplained variance (*χ*^2^ = 11.989, df = 2, *p* = 0.002). For logit ≤ 0, the case is NAD more likely than HCC. For logit > 0, the case is HCC more likely than NAD. Classifier based on this model is illustrated in Figure 5(a). The *y*-axis represents the linear combination of *z*_11_ and *z*_27_ in the above equation. The horizontal dotted line indicates the threshold level in the equation, 0.114, above which a lesion is classified as HCC and otherwise, NAD.

### 3.4. Augmented Predictor Model

The stepwise forward procedure stops at step 5 where the prediction accuracy is optimal, yielding the following regression:

(12) $$\begin{array}{c} logit=-0.586z_{1}^{\prime }+0.650z_{2}^{\prime }-3.72z_{11}+7.72z_{13}+13.9z_{25}-1.39 \end{array}$$

where *z*_1_′ and *z*_2_′ are the squares of sum of the ontological features and their absolute values, which were incorporated into the model in the first two steps; *z*_11_, *z*_13_, and *z*_25_ represent the activation values of “radiologic finding,” “mass of body region,” and “imaging result abnormal,” respectively, which were included in steps 3–5. The augmented predictors, *z*_1_′ and *z*_2_′, and the primary predictors, *z*_11_, “radiologic finding,” *z*_25_, and “imaging result abnormal” are significantly associated with the log-odds of HCC (*p* = 0.014, 0.006, 0.003, and 0.04). The constant has been also adjusted to compensate the imbalanced NAD and HCC cases. Omnibus test shows that the variance of log-odds explained by the model is significantly greater than the unexplained variance (*χ*^2^ = 70.619, df = 5, *p* < 0.001). For logit ≤ 0, the case is NAD more likely than HCC. For logit > 0, the case is HCC more likely than NAD. Classifier based on this model is illustrated in Figure 5(b). In step 5, the linear combination of the augmented predictors, *z*_1_′ and *z*_2_′, forms the *y*-axis and that of the primary predictors, *z*_11_, *z*_13_, and *z*_25_, the *x*-axis. The classifier is represented by the dotted line.

### 3.5. Performance Comparison of Models

Using the PPM, 98.1%, that is, 52 out of 53 NAD cases, and 57.6%, that is, 34 out of 59 HCC cases, are correctly classified. The overall accuracy is 76.8%. Using the APM, the correctly classified HCC cases increase significantly to 84.7% (*p* < 0.0001), which consist of 50 out of 59 HCC cases. Although the correctly classified NAD cases are reduced slightly to 92.5% (*p* = 0.250), the APM raises the overall accuracy to 88.4% significantly (*p* < 0.0001). The comparison of performances is summarized in Table 2.

## 4. Discussion

This study illustrated an approach for characterizing textual image reports by numerical values that weight the alignment of report contents with the ontological standard. Such approach has been demonstrated in our previous study where all the level 4 feature concepts of SNOMED-CT were considered to characterize the same set of image reports used in this study [22]. Using the specific term weighting, the highest overall accuracy, 74.3%, was attained for mapping report pairs based on the similarity measure of modified direction cosine. In this study, the features were further converted to standardized values, following *N*(0, 1), by inverse normalization [26]. Such conversion could help reduce the noise or outlier that was induced to the features through the edge-counting approach. The converted features were considered as primary predictors. Binary logistic regression model, identified using the primary predictors as the candidates in stepwise forward procedure, was used to classify the reports. The overall accuracy was increased to 76.8%.

It was shown that the interfeature association levels in HCC and NAD groups exhibited significantly different distributions where the feature concepts have particularly strong association in HCC [29]. This observation led to the derivation of two new features, which are squared sums of the existing features and their absolute values. We proved that the expected values of these two new features, which are estimated by their averages, represent the lower and upper limits of the sum of association levels over the group. The new features were combined with the existing features to provide the augmented predictor set for the stepwise forward procedure. It was found that the overall accuracy was significantly increased to 88.4% (*p* < 0.0001). The sensitivity, an important diagnostic performance indicator, was also significantly increased from 57.6% to 84.7% (*p* < 0.0001). Besides the first two new features, we identified the feature concepts: “radiologic finding,” “mass of body region,” and “imaging result abnormal.”

For new suspected cases, this panel of predictors representing a disease signature can be used to assist the clinical decision when associations of those pairs are observed. In future work, the discovered signature should be validated with independent data before its clinical applications. The detailed underlying meanings of the signature in patient management should be further explored using big data analytics.

An alternative application of the identified association patterns is the detection of inaccurate medical coding. When a disease is diagnosed, the “coactivated” feature concepts can be obtained and checked against the pairs in the disease-specific patterns. Potential inaccurate coding can be detected and the clinicians will be alerted. On a public health level, systematic failure in appropriate medical coding may result in under- or overadjustment to case-mix measurements when assessing quality of care [30]. In some healthcare models, this will also affect billing, reimbursement, and insurance claims [31].

Some observed image patterns mentioned in the image reports cannot be mapped to concepts in SNOMED-CT. For example, intravenous contrast injection induces changes of pixel optical density in different phases of CT scan. Contrast enhancement in particular phases is critically important for HCC diagnosis. However, SNOMED-CT has not defined the concepts, which could represent closely “contrast enhancement,” “arterial enhancement,” and “hyperdensity in arterial phase.” This is a limitation of this study that hindered the precision of the proposed predictor model.

## 5. Conclusions

This study demonstrated the extraction of ontological features from image report contents based on the ontological standard. Combining new features, derived from the differential association patterns, with the ontological features forms a panel of augmented predictors that signifies the HCC image reports.

## Figures and Tables

**Table 1:** Feature concepts and feature vectors of representative NAD and HCC cases.

Class	NAD	HCC
Abdominal organ finding	0	0.949
Blood vessel finding	0	0
Disorder of body cavity	−0.253	0.097
Disorder of body system	−0.140	−0.108
Disorder of cardiovascular system	0	0.319
Disorder of digestive system	−0.431	−0.399
Disorder of soft tissue	0	0.074
Disorder of trunk	−0.253	0.454
Finding of trunk structure	−0.253	1.165
Liver finding	0	0.349
Radiologic finding	0	−1.267
Cyst of abdomen	0	0
Mass of body region	0	0.502
Mass of digestive structure	0	0.502
Neoplastic disease	0	0
Growth alteration	0	0
Imaging result abnormal	0	0
Mechanical abnormality	0	−0.253
Finding of biliary tract	−0.842	0
Hemorrhage into peritoneal cavity	0	0
Disorder of connective tissue	0	0
Degenerative abnormality	0	0
Traumatic and/or nontraumatic injury of anatomical site	0	0
Abnormal radiologic density, diffuse	0	−0.431
Imaging result abnormal	0	−0.566
Abnormal radiologic density, irregular	0	0
Abnormal radiologic density, nodular	0	0
Abnormal radiologic density, small area	0	0
Multiple lesions	0	−0.674
Finding of number of lesions	0	−0.842

**Table 2:** Performances of primary predictor model (PPM) and augmented predictor model (APM) in classifying NAD and HCC cases. HCC and NAD predictions are indicated by “+” and “−,” respectively.

Model	Classification rate		Overall accuracy (112 cases)
	NAD (53 cases)	HCC (59 cases)	
PPM	98.1% (52 cases)	57.6% (34 cases)	76.8% (86 cases)
APM	92.5% (49 cases)	84.7% (50 cases)	88.4% (99 cases)
Contingency table	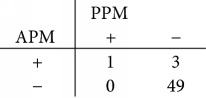	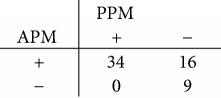	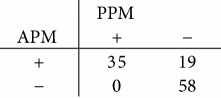
McNemar test	*p* = 0.250	*p* < 0.0001	*p* < 0.0001
